# Volumetric measurements of paranasal sinuses and examination of sinonasal communication in healthy Shetland ponies: anatomical and morphometric characteristics using computed tomography

**DOI:** 10.1186/s12917-021-02748-6

**Published:** 2021-01-21

**Authors:** Lisa Köhler, Ellen Schulz-Kornas, Ingrid Vervuert, Claudia Gittel, Karsten Winter, Dagmar Berner, Kerstin Gerlach

**Affiliations:** 1 Department for Horses, Faculty of Veterinary Medicine, University of Leipzig, An den Tierkliniken 21, D-04103 Leipzig, Germany; 2 Max Planck Institute for Evolutionary Anthropology, Max Planck Weizmann Centre for Integrative Archaeology and Anthropology (MPWC), Deutscher Platz 6, D-04103 Leipzig, Germany; 3 Department of Cardiology, Endodontics and Periodontology, University of Leipzig, Liebigstr. 12, D-04103 Leipzig, Germany; 4 University of Leipzig, Institute of Animal Nutrition, Nutrition Diseases and Dietetics, (IAND), , An den Tierkliniken 9, D-04103 Leipzig, Germany; 5 Queens Veterinary School Hospital, University of Cambridge, Madingley Road, Cambridge, CB3 0ES UK; 6 Institute of Anatomy, University of Leipzig, Liebigstraße 13, D-04103 Leipzig, Germany; 7 Equine Referral Hospital, Royal Veterinary College, University of London, Hawkshead Lane, Leipzig, Hertfordshire AL9 7TA UK

**Keywords:** Computed tomography, Paranasal sinuses, Shetland ponies, Sinonasal channels

## Abstract

**Background:**

Despite clinical importance and frequent occurrence of sinus disease, little is known about the size of paranasal sinuses and their communication in ponies and small horses. To examine the shape and volume of the paranasal sinuses and evaluate the sinonasal communication, three-dimensional (3D) reconstructions of computed tomography (CT) datasets of 12 healthy adult Shetland ponies were performed and analysed. Linear measurements of head length and width were taken. Using semi-automatic segmentation, 3D-models of all sinus compartments were created. Volumetric measurement of the seven sinus compartments were conducted and statistical analysis was performed. Sinus volumes were compared between the left and right sinuses and the relation to age and head size was evaluated.

**Results:**

Structure and shape of the paranasal sinus system in Shetland ponies was similar to that of large horses. All seven sinus compartments on each side of the head were identified (rostral maxillary sinus, ventral conchal sinus, caudal maxillary sinus, dorsal conchal sinus, middle conchal sinus, frontal sinus, sphenopalatine sinus). The existence of a bilateral cranial and a caudal system formed by a maxillary septum was visible in all 12 individuals. The volumetric sizes of the left and right sinuses did not differ significantly (*p* > 0.05). A positive correlation between the size of the paranasal sinuses and the head length was shown. A relation between sinus volumes and age could not be proved in adult ponies aged > six years. Communication between single sinus compartments was identified. Furthermore, communication with the nasal cavity over the nasomaxillary aperture (Apertura nasomaxillaris) and a common sinonasal channel (Canalis sinunasalis communis) as well as its splitting up into a rostral and a caudolateral channel could be seen. Examination of the sinonasal communication was challenging and only a descriptive evaluation was possible.

**Conclusions:**

Our findings concerning the size, shape and volumetric dimensions of Shetland pony CT images could help improve CT interpretation of abnormal clinical cases as well as aiding clinicians to develop and select appropriate instruments for medical inspection and treatments.

## Background

Equine paranasal sinuses are a complex bilateral system of air-filled cavities. On both sides of the head, you can find a cranial and a caudal system, which do not normally communicate, divided by a thin maxillary septum [1]. The cranial system consists of the rostral maxillary sinus (Sinus maxillaris rostralis, RMS) and the ventral conchal sinus (Sinus conchae ventralis, VCS). The caudal maxillary sinus (Sinus maxillaris caudalis, CMS), the dorsal conchal sinus (Sinus conchalis dorsalis, DCS), the middle conchal sinus (Sinus conchae medialis, MCS), the frontals sinus (Sinus frontalis, FS) and the sphenopalatine sinus (Sinus sphenopalatinus, SPS) belong to the caudal sinus system.

Within the systems there are communication ways between the belonging sinuses. The RMS and the VCS of the cranial system communicate via an opening dorsal to the nasolacrimal duct named the conchomaxillary aperture (Apertura conchomaxillaris) [2, 3]. The caudal sinus system consists of many communication channels. Due to the wide-open connection termed frontomaxillary aperture (Apertura frontomaxillaris) of the FS and DCS, both structures are often referred to as conchofrontal sinus (Sinus conchofrontalis). CMS and SPS communicate via the sphenopalatinal aperture [2, 3]. Additionally, there is a connection between the MCS and CMS [4], which according to literature has no proper scientific name.

The cavities of the 2 systems communicate with the middle meatus of the nasal cavity via the nasomaxillary aperture (Apertura nasomaxillaris) [2, 5]. The common sinonasal channel splits up into an rostral and a caudal sinonasal channel [2, 5]. The rostral one can be connected either to the RMS or to the VCS or to both sinuses [5]. The caudal one is connected to the CMS [2, 3, 5].

The three-dimensional structure and volume of paranasal sinuses in ponies and small breed horses is poorly described in the literature. To date, no data are available on volumetric measurements of the sinus system in ponies. There are several equine studies that have evaluated size and volumes of the paranasal sinuses in adult horses, providing reference values and finding a relation between sinuses volume and head dimension and between sinuses volumes and age [1, 3, 5–8]. Communication ways between paranasal sinuses themselves and with the nasal cavity are described and size measurements are given. Information concerning the relationship between the size of paranasal sinuses and head dimensions may help clinicians to obtain information on sinus volumes when planning surgery. Precise knowledge about the anatomical structure is essential for successful treatment of diseases in this region. Sinusitis caused by an infection of the teeth is the most common disease in horses, followed by primary sinusitis, trauma, progressive haematoma, paranasal cysts and neoplasia [9]. The compartments most affected by sinus disease are the CMS (78 %) and the RMS (61 %) [10].

An accepted and very important diagnostic method for structures of the equine head is computed tomography [9, 11–13].

Our hypothesis was that the anatomical findings in Shetland ponies are in accordance with large horse breeds but the sinus volumes are smaller. The aim of the study was to describe the CT anatomy of paranasal sinuses and communication ways in adult ponies. Additionally, volumetric measurements of the paranasal sinuses were performed and the relations between sinus volumes and head size were evaluated.

## Results

**Fig. 1:**
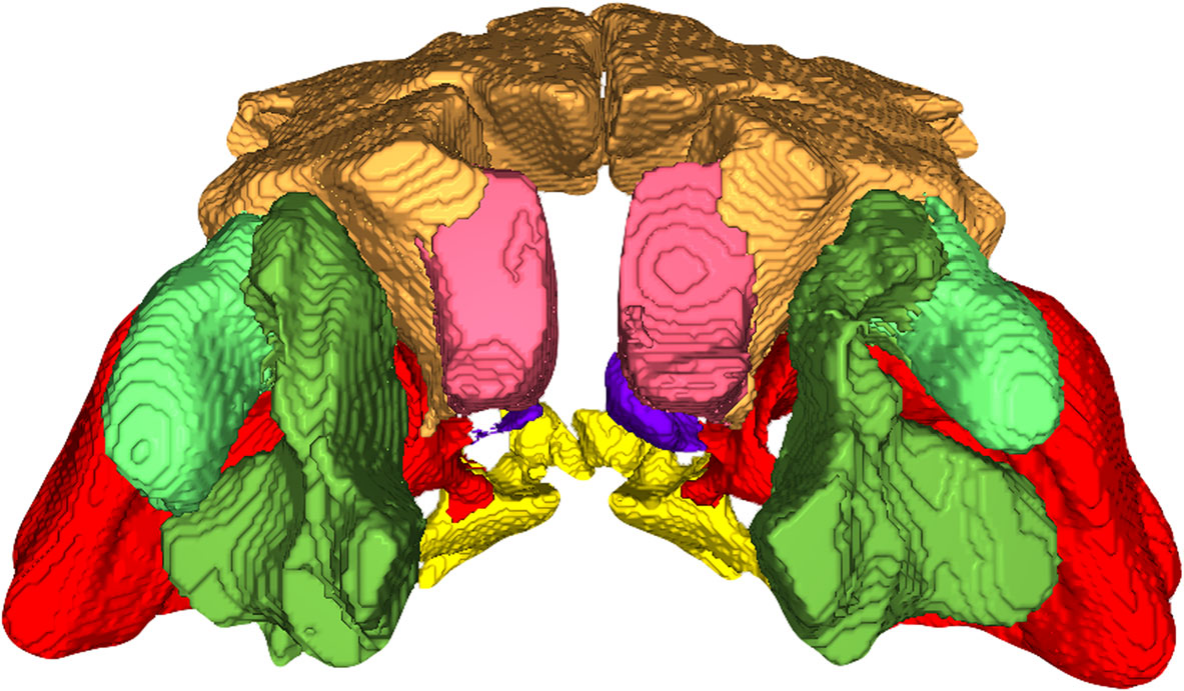
3D-model by CT-reconstruction of all paranasal sinuses in a rostral view. Paranasal sinuses of a six-years old male Shetland pony. Coloured structures: dark green: Ventral conchal sinus, light green: Rostral maxillary sinus, red: Caudal maxillary sinus, orange: Frontal sinus, pink: Dorsal conchal sinus; violet: Middle conchal sinus, yellow: Sphenopalatine sinus

**Fig. 2:**
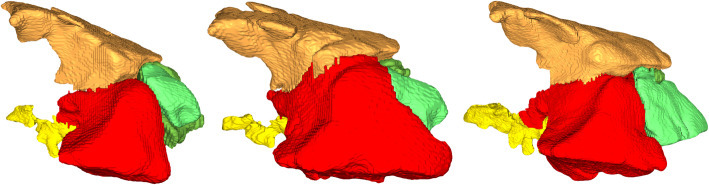
3D-models by CT-reconstruction of sinus compartments of the right side (lateral view) of 3 ponies. Individual size and shape of single sinus compartments can be seen in these figures of 3 different adult ponies **a** male, 7 years; **b** male,14 years and **c** unknown gender, 25 years. Coloured structures: dark green: Ventral conchal sinus, light green: Rostral maxillary sinus, red: Caudal maxillary, orange: Frontal sinus, violet: Middle conchal sinus, yellow: Sphenopalatine sinus

Shetland ponies of both sexes (5 males, 3 females, 2 of unknown gender) aged from six to 25 years (mean age 11.75 years) were examined. Weights ranged between 95 kg and 149 kg (mean weight 130.2 kg). In every individual, all seven paranasal sinuses (Fig. 1) could be identified on the right and the left side. Inter- and intraindividual differences in size and shape (Fig. 2) were identified, but location and basic shape defined by the surrounding bony structures were the same. The rostral maxillary sinus was the most rostral sinus compartment in all ponies. In two different individuals out of a total of 12 scans, the caudal maxillary sinus on the right side bulged out and overhung the rostral maxillary sinus rostrally. Both maxillary sinuses were separated by the maxillary septum, which was incomplete on the right side in one pony. Medial to the rostral maxillary sinus, the ventral conchal sinus, separated by a thin bony septum or its imaginary continuation, extended rostrally. This bony septum was oriented obliquely to the nasolacrimal duct and was incomplete, allowing for communications between these two sinus compartments. Situated caudolaterally to the rostral maxillary sinus, the caudal maxillary sinus was connected to the frontal sinus dorsally and to the branched sphenoplatine sinus caudoventrally. The middle conchal sinus was situated caudomedially between the openings of the frontal and sphenopalatine sinus. The large frontal sinus had a wide opening to the dorsal conchal sinus, which was located ventromedially.

**Fig. 3:**
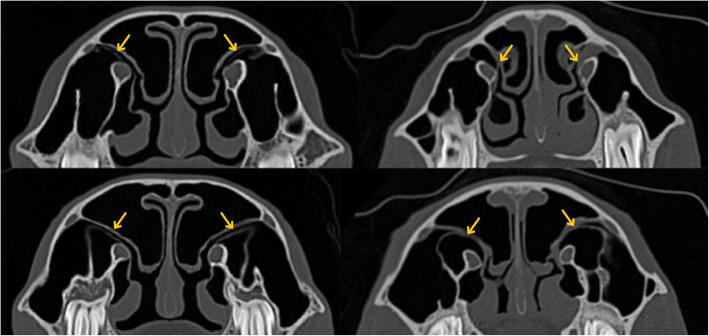
Sinonasal communication channels between nasal cavity and Rostral maxillary (**a**) and Caudal maxillary sinus (**b**). Continuous communication can be seen on the left side, but not on the right side (yellow arrows)

Communication between the paranasal sinuses was present in every individual: conchomaxillary aperture, frontomaxillary aperture and sphenopalatinal aperture. In one pony, there was no communication found between CMS and MCS. Another pony showed this connection only on the right side. Communication channels between the nasal cavity and the maxillary sinuses could not be identified in every case (Fig. 3) and showed many variations (Table 1).

**Table 1:** Presence and number of visible entrances from nasal cavity to rostral and caudal maxillary sinus (*N* = 12)

Pony number	Rostral nasomaxillary aperture		Caudal nasomaxillary aperture	
	left	right	left	right
1	+	-	-	-
2	+	++	-	-
3	-	-	+	+
4	+	+	-	+
5	-	-	-	-
6	+	++	+	-
7	+	+	+	+
8	-	-	-	-
9	+	-	-	-
10	++	++	-	-
11	-	-	-	-
12	+	-	+	-

- no entrance, + one entrance, ++ split entrance

**Fig. 4:**
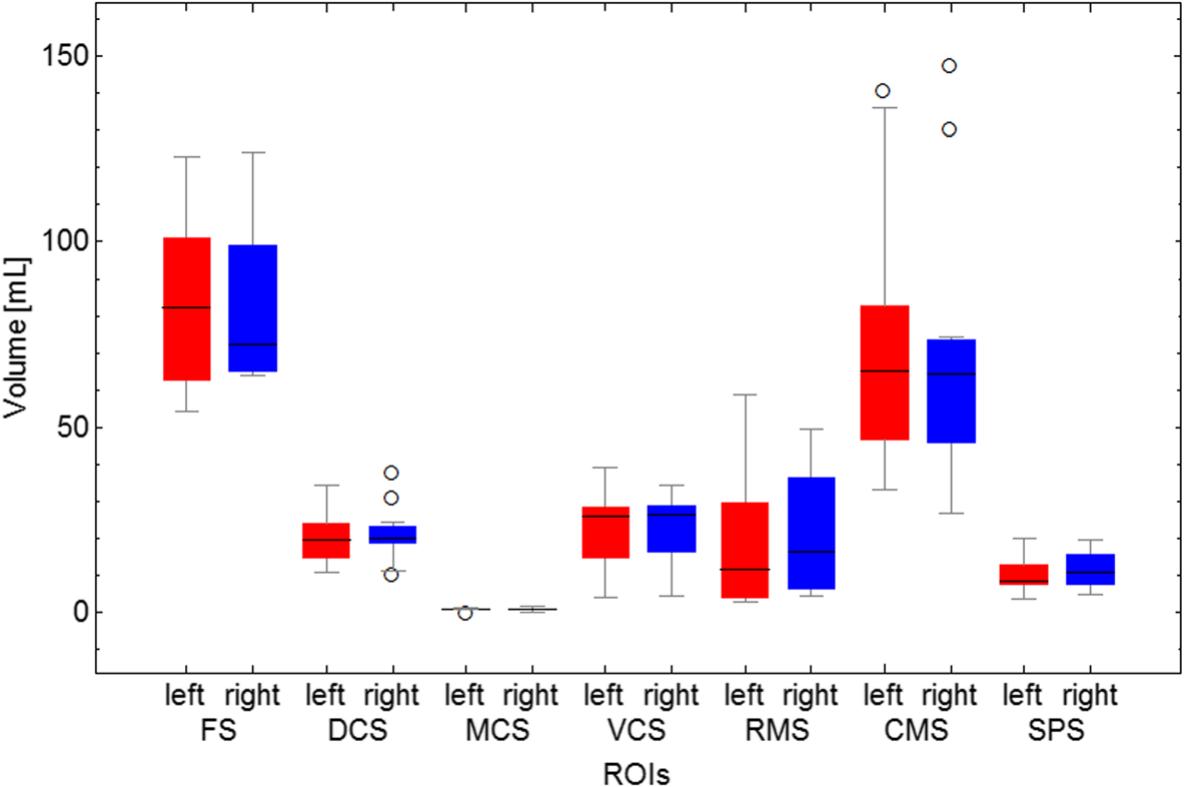
Boxplots of regions of interest (ROI): sinus volumes for left and right side (*N* = 12). Centre line: median, whiskers: minimum and maximum intervals, box: 25/75 percentile, circles: mild outliers, asterisk: extreme outliers, mL: millilitre, ROI: Region of interest, FS: Frontal sinus, DCS: Dorsal conchal sinus, MCS: Middle conchal sinus, VCS: Ventral conchal sinus, RMS: Rostral maxillary sinus, CMS: Caudal maxillary sinus, SPS: Sphenopalatine sinus

Total sinus volumes ranged between 275.26 and 822.62 mL (mean = 459.66 mL). When comparing the left and right side, total volumes, volumes of rostral and caudal system and volumes of single sinus compartments showed no significant differences (*p* < 0.05). The volumes are displayed in Table 2 and in Fig. 4.

**Table 2:** Absolute volumes of all sinus compartments, rostral and caudal system (*N* = 12)

		Mean	SD	Min	Max	Median	Q25	Q75	IQR
**Total (mL)**	total	459.66	33.77	275.26	822.62	438.43	348.94	482.75	133.81
	left	230.13	34.52	124.78	417.78	219.82	173.47	244.81	71.34
	right	229.52	33.21	145.16	404.84	209.21	177.13	238.18	61.05
**Rostral System (mL)**	total	85.73	13.79	45.56	171.55	79.05	63.17	87.8	24.63
	left	41.94	14.79	16.46	98.11	35.78	29.41	47.73	18.32
	right	43.79	13.03	25.07	83.89	38.38	34.07	44.4	10.33
**Caudal system (mL)**	total	373.92	38.11	266.61	651.07	359.13	276.38	401.33	124.95
	left	188.19	38.87	108.33	319.67	175.58	133.66	214.78	81.3
	right	185.73	37.65	118.29	331.4	173.56	136.94	199.21	62.27

Total number of sinus compartments, rostral and caudal systems, also divided into left and right side in Shetland ponies (n=12); *SD*: standard deviation, *Min*: minimum, *Max*: maximum, *Q25*: 25th percentile, *Q75*: percentile, *IQR*: interquartile range, *mL*: millilitre

The head length ranged from 35.07 cm to 43.35 cm (mean 38 cm) and the head width ranged from 14.12 cm to 17.06 cm (mean 14.9 cm).

Correlations between single sinus volumes and head length and head width differed. A positive correlation between the total volume of the paranasal sinuses and the volumes of FS, DCS, CMS, SPS with the head length was found. There were no correlations between the total volume of the sinuses and the head width. Only the volume of the RMS showed a positive correlation to the head width. MCS and VCS showed no relation to the head length or width.

Significant correlations between sinus volumes and age were not found.

## Discussion

CT-imaging with three-dimensional reconstruction proved an appropriate method for this study. Correct identification and demarcation of the paranasal sinuses is very challenging because of the complex anatomy. In addition to radiographic imaging and transnasal endoscopy, computed tomography has become an established diagnostic method for sinus disease [12, 13]. Without superimposition of anatomical structures [12, 13] and the possibility of three-dimensional reconstruction [13], it provides detailed and valuable information for correct diagnosis and surgical planning [12].

Our findings concerning number, position and basic shape of paranasal sinuses proved a high similarity to large horses.

Openings connecting different sinuses were present in every head and were clearly identifiable. A connection between CMS and MCS, which is described in literature [4], was not visible in one pony. Another individual showed this connection only on one side. We cannot rule out that previous disease might have caused changes in this connection.

Identifying sinonasal communication channels is already described for large horses [5, 6, 14], but proved very difficult in the present study and was not found for every pony. Due to the high variations in the position of surrounding structures, a reliable measurement was impossible. The small size alone may be one reason. Mucosal swelling during anaesthesia [13] and partial volume effect are known artefacts and may affect the presentability of the sinonasal communication channels. Post-mortem changes and the absence of soft tissue in the head from the abattoir and the skull might have the effect of better visibility. However, even there, it was not possible to identify access from the nasal cavity to both maxillary sinuses. An additional study using gross dissection and macroscopic assessment would be useful to clarify and compare our findings of the anatomical structures in order to determine whether sinonasal communication exists.

Volume measurements have been taken in large horses [1, 7, 8]. Based on a previous equine study by Brinkschulte et al. [5], using semiautomatic segmentation for volumetric measurements of paranasal sinuses, data obtained in the present study allow comparison between Shetland ponies and horses. Indeed, the number and structure of paranasal sinuses found to be equivalent in for horses were confirmed in the present study. Exact separation of the paranasal sinuses depends on the examiner’s experience, this being challenging and also subjective.

In general, sinus volumes in Shetland ponies are smaller than in large horses, being usually more than half the size of horses. Similar to horses, there was no significant difference between the left and right side in ponies [1, 7]. Data of the single head and the skull, which underwent scans post mortem did not differ significantly from data obtained from living individuals. The absence of soft tissue does not influence measurements. Nevertheless, other post-mortem changes may entail alterations in structure and shape of the bones, especially in the skull of the bone exponent, and should be considered as a limitation of the study.

A positive relation between head size and sinus volumes was found for large horses [7, 15]. Liuti et al. [7] found a positive correlation between sinus volumes and head volume, taking measurements of a length from the caudal aspect of the orbit to the nasoincisive notch in a sagittal reconstruction [16]. Brinkschulte [15] found a positive correlation for a head length measured from the crown of an incisive to the occiput in a reconstructed lateral view. In our study, we used validated measurement methods from Froydenlund (2005) [17] and Evans & McGreevy [18]. Although different methods for length measurement were used in literature, relations between the head sizes and sinus volumes were similar and another conformity among adult equids was shown.

Nevertheless, it is known that intraspecific differences in head shapes of domesticated horses are great [19]. Studies about conformation [18] and shape variation [19] of the equine skull describe differences between various horse breeds. With the varying shape of cranial profiles, the values of cranial and nasal portions differ. A concave profile has higher nasal values than a convex profile [18]. The profile of ponies tend to be concave in smaller breeds including Shetland ponies, with broad frontal bones and broad to normal zygomatic bars, whereas convex nasal bones with broad frontal bones are found in draught horses, and concave nasals with narrow frontals in light horses [19]. The disparity among different breeds suggests that ponies are not small horses. This might be the reason as to why a correlation between sinus volumes and head width was present in studies by Liuti et al. [7] and Brinkschulte [15], but not in ponies in the present study.

In contrast to other authors[1, 7] who describe age-related increasing sinus volumes, there was no significant correlation found among the present group of adult ponies. Young growing ponies were excluded from our study. It would be promising to conduct future studies using higher numbers of individuals of various ages to test for ontogenetic influences.

## Conclusions

Paranasal sinuses are visualisable and evaluable similar to large horses. Computed tomography, semiautomatic segmentation and three-dimensional reconstruction make precise anatomical evaluation and reliable size-measurement possible. The visualisation of sinonasal communication pathways is challenging and cannot be identified for every pony via CT. Our findings concerning the relation between paranasal sinus volumes to head sizes and age differ from those described for large horses in literature. The results of this study may help clinicians to successfully treat sinus disease in ponies.

## Methods

Computed tomography (CT) was performed on ten different heads of adult Shetland ponies. Two of them were examined twice at different ages so that we were able to include 12 scans in our study. Seven ponies were included in one of two research studies with different aims. This research was approved by the Ethics Committee for Animal Rights Protection of the Leipzig District Government (No. TVV 05/15 and 32/15) in accordance with German legislation on Animal Rights and Welfare. After completion of the project, all ponies were moved to the University of Vienna, Austria to be included in further research studies.

One individual was a client-owned clinical case, one head was provided by the Institute of Veterinary Pathology of the University of Leipzig, Germany and one head was a skull, a bone specimen from the Institute of Veterinary Anatomy of the University of Leipzig, Germany. The examinations of the healthy ponies were performed under general anaesthesia in dorsal recumbency. The examinations of the single head and the skull were performed in ventral recumbency post-mortem. Criteria for case selection were the breed (Shetland pony) and the absence of a known history or clinical findings of paranasal sinus disease. The study was designed as a descriptive, retrospective cross-sectional study.

The acquisition of the images was performed at the Department for Horses of the Veterinary Faculty of the University of Leipzig, Germany using a multi-detector row CT unit (Mx8000 IDT 16 CT scanner, Philips Medical Systems DMC GmbH, Hamburg, Germany). The following settings were used during image acquisition: a tube voltage of 120 kV, tube current (200mAs), 0.75 second tube rotation time, pitch (0.438) and 1 mm slice thickness without a gap or overlap. After setting the window width at -500 HU (Hounsfield units) and the window level at 2000 HU with a 512 × 512 matrix, images were taken.

These were reconstructed three-dimensionally (3D) (Philips CT-Software, Philips Medical Systems DMC GmbH, Hamburg, Germany) in dorsal projection, positioned orthogonally to the bony palate by one of the authors (KG). Head length was measured in accordance with Froydenlund (2005) from the premaxilla to the occiput, and the head width in accordance with Evans and McGreevy [18] “at the level of the nasal palpebral commissure” in reconstructed images by another author (LK).

The CT-images were analysed slice by slice in transverse, dorsal and sagittal planes and the paranasal sinuses, anatomical characteristics and connective junctions among the paranasal sinuses and with the nasal cavity were evaluated (LK).

For segmentation and volume rendering of the paranasal sinuses, the program CIBC Seg3D 2 Segmentation (Version 2.4.4; NIH/NIGMS CIBC; https://www.seg3d.org/) and the reconstructed transversal CT-datasets were used. Semiautomatic segmentation was performed. For this purpose, a tissue line was created using a mask from − 1024 to -600 HU (Hounsfield units) to segregate two tissues. A seed point was set in every slice manually and a 2D-growing algorithm was performed. Afterwards, manual correction was performed slice by slice. Volumes were computed and an unconstrained smoothing algorithm was applied to smoothen the surfaces. Using volume rendering, 3D models of the sinuses and sinonasal channels were created and sinuses volumes were measured (Figs. 1 and 2). The whole procedure was performed in accordance with Brinkschulte et al. [1].

### Data analysis

Data collection was performed using a spreadsheet (Excel® office 2019, Microsoft® Corporation, Redmond, WA, USA). For statistical analysis, the program Mathematica® (Version 12.0, Wolfram Research Inc., Champaign, IL, USA) was used. Descriptive statistics were computed and box plots were generated.

Using the Shapiro-Wilk test, volumes of the left and right sides were tested for normal distribution. The paired sample t-test was performed for normally distributed data and the Wilcoxon test for non-normally distributed data.

Correlation between volumes and age, head length and head width were calculated using Spearman’s rank correlation coefficient. Significance for all tests was set at *p* < 0.05.

## Data Availability

The datasets used and analysed during the current study are available from the corresponding author on reasonable request.

## Abbreviations

2D: Two-dimensional
3D: Three-dimensional
CMS: Caudal maxillary sinus
CT: Computed tomography
DCS: Dorsal conchal sinus
FS: Frontal sinus
HU: Hounsfield units
IQR: Interquartile range
MCS: Middle conchal sinus
Min: Minimum
Max: Maximum
Q25: 25^th^ percentile
Q75: 75^th^ percentile
RMS: Rostral maxillary sinus
SD: Standard deviation
SPS: Sphenoplatine sinus
VCS: Ventral conchal sinus

## References

[1] Brinkschulte M, Bienert-Zeit A, Lüpke M, Hellige M, Staszyk C, Ohnesorge B (2013). Using semi-automated segmentation of computed tomography datasets for three-dimensional visualization and volume measurements of equine paranasal sinuses. Vet Radiol Ultrasound.

[2] Nöller C, Nowak M, Hamann J, Fritsch G, Budras K-D (2007). Anatomy of the equine nasal cavity and paranasal sinuses and potential clinical applications as a basis for endoscopy, computed tomography and surgery. Pferdeheilkunde.

[3] Probst A, Henninger W, Willmann M (2005). Communications of normal nasal and paranasal cavities in computed tomography of horses. Vet Radiol Ultrasound.

[4] Gerhards H, Huskamp B, Deegen E, Wissdorf H (2011). Praxisorientierte Anatomie und Propädeutik des Pferdes: Naseneingang, Nasenhöhle und Nasennebenhöhlen.

[5] Brinkschulte M, Bienert-Zeit A, Lüpke M, Hellige M, Ohnesorge B, Staszyk C (2014). The sinonasal communication in the horse: examinations using computerized three-dimensional reformatted renderings of computed-tomography datasets. BMC Vet Res.

[6] Tatarniuk DM, Bell C, Carmalt JL (2010). A description of the relationship between the nasomaxillary aperture and the paranasal sinus system of horses. Vet J.

[7] Liuti T, Reardon R, Dixon PM (2017). Computed tomographic assessment of equine maxillary cheek teeth anatomical relationships, and paranasal sinus volumes. Vet Rec.

[8] Bahar S, Bolat D, Dayan MO, Paksoy Y (2014). Two- and Three-Dimensional Anatomy of Paranasal Sinuses in Arabian Foals. Vet Med Sci.

[9] Feige K, Geissbühler U, Fürst A, Ehrat F, Schwarzwald C (2000). Paranasal sinus disease in horses: a retrospective study of 55 cases. Pferdeheilkunde.

[10] Dixon PM, Parkin TD, Collins N, Hawkes C, Townsend N, Tremaine WH (2012). Equine paranasal sinus disease: a long-term study of 200 cases (1997–2009): ancillary diagnostic findings and involvement of the various sinus compartments. Equine Vet J.

[11] Henninger W, Mairi Frame E, Willmann M, Simhofer H, Malleczek D, Kneissl SM, Mayrhofer E (2003). CT features of alveolitis and sinusitis in horses. Vet Radiol Ultrasound.

[12] Kinns J, Pease A (2009). Computed tomography in the evaluation of the equine head. Equine Vet Educ.

[13] Solano M, Brawer RS (2004). CT of the Equine Head: Technical Considerations, Anatomical Guide, and Selected Diseases. Clin Tech Equine Pract.

[14] Morrow KL, Park RD, Spurgeon TL, Stashak TS, Arceneaux B (2000). Computed tomographic imaging of the equine head. Vet Radiol Ultrasound.

[15] Brinkschulte M (2014). Morphologische Untersuchung der Apertura nasomaxillaris des Pferdes sowie deren Verzweigung in die Nasennebenhöhlen unter Anwendung dreidimensionaler Rekonstruktion computertomographischer Schnittbildserien.

[16] Liuti T, Reardon R, Smith S, Dixon PM (2016). An anatomical study of the dorsal and ventral nasal conchal bullae in normal horses: Computed tomographic anatomical and morphometric findings. Equine Vet J.

[17] Froydenlund TJ, Dixon PM, Smith SH, Reardon RJM. Anatomical and histological study of the dorsal and ventral nasal conchal bullae in normal horses. Vet Rec. 2015;177:542. 10.1136/vr.103408. 10.1136/vr.103408 26585864

[18] Evans KE, McGreevy PD (2006). Conformation of the equine skull: a morphometric study. Anat Histol Embryol.

[19] Heck L, Wilson LAB, Evin A, Stange M, Sánchez-Villagra MR (2018). Shape variation and modularity of skull and teeth in domesticated horses and wild equids. Front Zool.

